# Acute respiratory distress syndrome: potential of therapeutic interventions effective in treating progression from COVID-19 to treat progression from other illnesses—a systematic review

**DOI:** 10.1136/bmjresp-2022-001525

**Published:** 2023-09-01

**Authors:** Emma J Ragel, Lynda K Harris, Richard A Campbell

**Affiliations:** 1 Division of Pharmacy and Optometry, University of Manchester, Manchester, UK; 2 Maternal and Fetal Health Research Centre, University of Manchester, Manchester, UK; 3 3St Mary’s Hospital, Manchester University NHS Foundation Trust, Manchester, UK; 4 Olson Center for Women's Health, University of Nebraska Medical Center, Omaha, Nebraska, USA

**Keywords:** COVID-19, ARDS, Respiratory Infection

## Abstract

**Background:**

Acute respiratory distress syndrome (ARDS) is the most severe form of lung injury, rendering gaseous exchange insufficient, leading to respiratory failure. Despite over 50 years of research on the treatment of ARDS when developed from illnesses such as sepsis and pneumonia, mortality remains high, and no robust pharmacological treatments exist. The progression of SARS-CoV-2 infections to ARDS during the recent global pandemic led to a surge in the number of clinical trials on the condition. Understandably, this explosion in new research focused on COVID-19 ARDS (CARDS) rather than ARDS when developed from other illnesses, yet differences in pathology between the two conditions mean that optimal treatment for them may be distinct.

**Aim:**

The aim of the present work is to assess whether new therapeutic interventions that have been developed for the treatment of CARDS may also hold strong potential in the treatment of ARDS when developed from other illnesses. The study objectives are achieved through a systematic review of clinical trials.

**Results:**

The COVID-19 pandemic led to the identification of various therapeutic interventions for CARDS, some but not all of which are optimal for the management of ARDS. Interventions more suited to CARDS pathology include antithrombotics and biologic agents, such as cytokine inhibitors. Cell-based therapies, on the other hand, show promise in the treatment of both conditions, attributed to their broad mechanisms of action and the overlap in the clinical manifestations of the conditions. A shift towards personalised treatments for both CARDS and ARDS, as reflected through the increasing use of biologics, is also evident.

**Conclusions:**

As ongoing CARDS clinical trials progress, their findings are likely to have important implications that alter the management of ARDS in patients that develop the condition from illnesses other than COVID-19 in the future.

**WHAT IS ALREADY KNOWN ON THIS TOPIC**

- The COVID-19 pandemic led to a huge increase in clinical trials on patients with acute respiratory distress syndrome (ARDS). ARDS developed from COVID-19 and from other illnesses have distinct pathologies, with dysregulated immune responses in the former, leading to hyperinflammation and cytokine storms.

**WHAT THIS STUDY ADDS**

- The systematic review evaluates the potential of novel therapeutic interventions developed for COVID-19 ARDS in the treatment of ARDS when developed from other illnesses. It is shown that complement system modifiers, cell-based therapies and biologics hold clear potential.

**HOW THIS STUDY MIGHT AFFECT RESEARCH, PRACTICE OR POLICY**

- It is hoped that the outcome will focus future clinical research on these interventions with potential benefits for patients with a range of conditions, including pneumonia and sepsis.

## Introduction

The COVID-19 pandemic has shone light on the inadequate treatment options for acute respiratory distress syndrome (ARDS). Within the United Kingdom (UK), 12.5% of intensive care unit admissions meet ARDS criteria,^1^ and in the United States of America (USA), ARDS affects around 150 000^2 3^ to 200 000^4^ people annually. ARDS is the most severe form of acute lung injury (ALI)^2 5 6^ and is characterised by inflammatory damage to the alveolar-capillary membrane, increasing capillary permeability,^7–11^ leading to oedema in the alveoli and lung interstitium.^7 11^ The initial damage can be direct *via* pneumonia, pulmonary infection or aspiration, or indirect *via* sepsis, drug toxicity, blood transfusions or non-pulmonary major trauma.^7–9 11 12^ Following activation of a local inflammatory response, the release of proinflammatory cytokines promotes further damage to the lungs.^6–10 12^

The resulting decrease in efficacy and capacity for gaseous exchange^13 14^ is worsened by alveolar atelectasis.^5 7 11–15^ Where ARDS has advanced to the fibrotic stage,^16 17^ surfactant production is impeded, encouraging atelectasis.^5 7–9 11–15^ Despite physiological attempts to correct hypoxemia and hypercapnia presenting as dyspnoea, the condition deteriorates to refractory hypoxemia and respiratory failure, necessitating mechanical ventilation (MV).^2 18–20^ MV incurs additional risks, including ventilator-induced lung injury (VILI) (eg, barotrauma),^5 10 21^ and an increased risk of nosocomial infections, particularly ventilator-associated pneumonia.^20^ Prolonged MV increases the incidence of neuromuscular dysfunction, potentially triggering multiple-organ dysfunction.^20^ Other ARDS complications include mood disturbances^16 20^ and organ dysfunction or failure.^22^ The leading cause of death is multiorgan failure,^1 8 10 20 22^ followed by thromboembolic complications^22^ and sepsis.^20^

The widely heterogenous nature of ARDS has contributed to a lack of effective pharmacological treatments, although identifying the initial clinical insult can aid the selection of therapies.^8 9^ Neuromuscular blocking agents (NMBAs) boost the lung and chest wall compliance, improving patient-ventilator synchrony and the extent of hypoxaemia, reducing oxygen consumption and the risk of VILI. In patients with severe ARDS, early administration of an NMBA for 48 hours improved patient survival and reduced ventilator dependence without causing muscle weakness.^23^ However, neuromuscular weakness can develop with their prolonged use, resulting in difficulties weaning patients off MV.^10 19^ Vasodilators are hypothesised to improve gaseous exchange relieving severe hypoxia, but therapy needs to be kept short term,^10 12^ and it is accepted that there is no mortality benefit.^24^ Bronchodilators may prevent worsening of alveolar-capillary permeability, helping to prevent further existing oedema, alongside offering potential anti-inflammatory effects,^10 13^ but studies have shown that they can be ineffective or even harmful in the treatment of ARDS.^25–27^ Glucocorticoids reduce inflammation and improve oxygenation and MV duration, but no significant changes in mortality rates have been reported, and their immunosuppressive actions may prolong viral replication.^10 12 18 19 22 28^ Despite the reported pleiotropic anti-inflammatory and antiproliferative effects of statins, their clinical benefit in ARDS remains inconsistent.^16 18 29^ Although surfactants do not improve mortality or MV duration in ARDS, they are thought to improve the mechanical properties of alveoli and, therefore, benefit CARDS patients, where clinical presentation is similar to infantile respiratory distress syndrome in which surfactants are routinely used.^15 16 18^ Alternative approaches include novel therapeutics such as mesenchymal stem cells (MSCs), which release bioactive factors promoting repair of injured lung tissue.^16^ However, despite the wide range of therapeutics available, morbidity and mortality rates remain high.

COVID-19-induced ARDS (CARDS) is the most severe clinical manifestation following infection with the SARS-CoV-2 virus.^30^ Soon after the start of the pandemic, and prior to global vaccination drives, COVID-19 infection became the most common cause of ARDS worldwide, with cases doubling in the USA,^31^ causing a marked increase in demands on healthcare services and ventilators worldwide and emphasising the need for new treatments.^31–33^

CARDS was initially managed similarly to ARDS,^34^ yet their differing pathologies mitigate the effectiveness of ARDS treatments in CARDS.^35–39^ In CARDS, dysregulated immune responses^30 38 40^ cause exaggerated increases in immune cells and inflammatory markers, particularly in severe COVID-19 infections,^30 38 40 41^ leading to hyperinflammation^38 40 42^ and cytokine storms.^32 38 40–44^ Cytokine storms, which are also observed in ARDS, are considered to be the main cause of CARDS^45^ and are linked to its progression with cytokine-neutralising agents, including interleukin-6 (IL-6) and IL-6 receptor inhibitors being used to clinical benefit.^38 40^ CARDS is more likely to require longer MV durations^30 32 33^ and potentially a higher VILI frequency,^43 46^ since prolonged MV is associated with persistently raised immune cells.^30^ Furthermore, CARDS has a higher incidence of intravascular thrombosis and other thromboembolic manifestations,^31 39^ which may be due to coagulation dysfunction.^39^ The distinctive clinical features of the conditions are summarised in table 1.

**Table 1:** Comparison of the distinctive clinical features of ARDS and CARDS^20 22 30 39^

Feature	ARDS	CARDS
Onset	Condition develops within 1 week of a clinical insult according to the Berlin definition^18 33^	Condition develops 8–12 days after onset of COVID-19 symptoms
Development	Neutrophil activation is key for ARDS development; the release of proinflammatory cytokines and chemokines cause further damage to the lungs; cytokine storm is associated with ARDS progression	Dysregulated systemic immune responses cause exaggerated increases in immune cells and inflammatory markers in the lung, particularly in severe infections, which leads to hyperinflammation and cytokine storm, which is the most common cause of CARDS
Mechanical intervention	Most ARDS patients require mechanical ventilation, with a median duration of 6–25 days	CARDS is characterised by normal or high lung compliance, requiring a longer duration of mechanical ventilation, leading to an increased incidence of VILI
Pathophysiology	ARDS is the outcome of a variety of different insults, leading to variation in symptoms, pathophysiology, potential therapeutic targets and disease severity in patients	CARDS is caused by a single clinical insult, so the underlying pathophysiology and clinical presentation are less variable than ARDS
Complications	Thromboembolic complications are a common cause of death	Increased coagulation dysfunction may be the underlying cause of the increased incidence of intravascular thrombosis and other thromboembolic complications reported
Mortality	Mortality is estimated to be 40%	Mortality was estimated to be almost 50% at the start of the pandemic but this has significantly reduced following vaccinations

ARDS, acute respiratory distress syndrome; CARDS, COVID-19 ARDS; VILI, ventilator-induced lung injury.

The COVID-19 pandemic has stimulated a rapid expansion in the number of clinical trials (CTs) assessing new interventions for CARDS. This presents a unique opportunity to evaluate whether new treatments initially developed for CARDS hold potential for use in the treatment of ARDS developed from other illnesses. The overarching aim of this study is to assess the potential of interventional treatments for CARDS in the treatment of ARDS from other illnesses through a systematic review of CTs on therapeutic interventions for ARDS and CARDS in the USA and UK. Highly valuable reviews have been published recently on a comparison of ALI in COVID-19 and non-COVID-19 patients,^47^ on the epidemiology of ARDS before and after pandemic,^48^ on current treatment for CARDS patients^32^ and on how atypical CARDS is in comparison with ARDS.^49^ However, to the knowledge of the authors, the present review represents the first with a scope to learn what strategies from the vigorous development of CARDS treatments hold potential for use in the treatment of ARDS from other illnesses.

## Methodology

### Study design

This retrospective, observational, systematic review of CTs was undertaken using the database ClinicalTrials.gov, which is provided by the US National Library of Medicine. This database was chosen because it includes publicly and privately funded research studies, allowing a comprehensive view of treatments being investigated.

### Database searches

**Figure 1:**
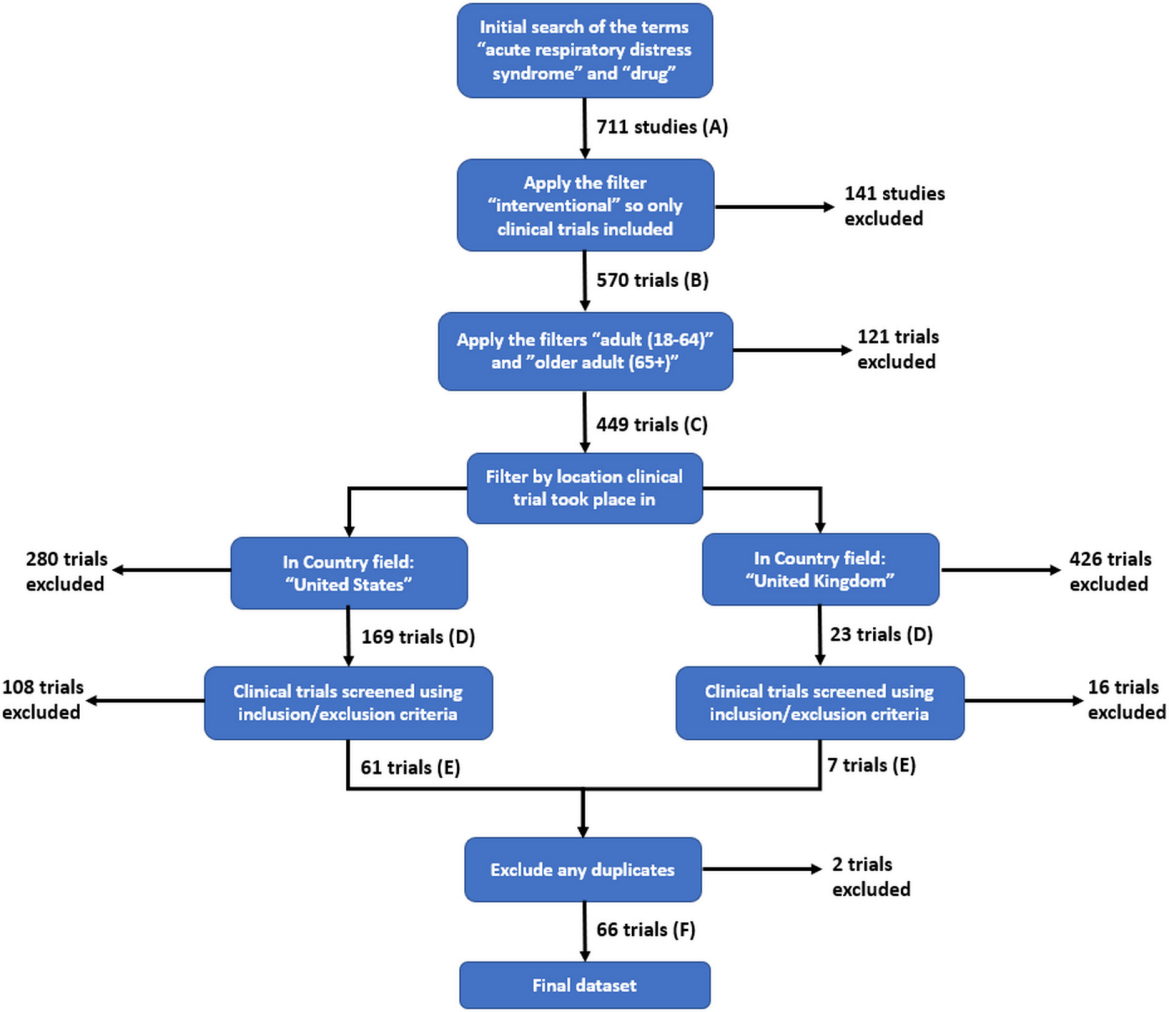
Workflow for CT identification illustrating the results obtained and the refinement process: (A) the number of CTs identified in the initial searches, (B–E) the numbers of CTs left following the application of defined exclusion criteria (see text for details), and (F) the number of CTs in the final dataset following the exclusion of duplicates.

All searches were carried out between 4 and 9 March 2022 using the search term ‘acute respiratory distress syndrome’ under the ‘condition or disease’ field, and ‘drug’ under the ‘other terms’ field, which identified 711 studies (figure 1A). This search was kept deliberately broad in order to include all related studies in the results, even if referred to in the title using synonyms of the conditions, including ALI.

Restriction of the data set to include only interventional studies reduced the number of trials to 570 (figure 1B). This step excluded studies that were expanded access or compassionate use, observational or behavioural, as they did not assess administration of a pharmacological agent.

Search criteria were further modified by selecting only ‘adult (18–64)’ and ‘older adult (65+)’ to ensure only participants aged 18 years or over were included, identifying 449 CTs (figure 1C); there are differences in disease pathophysiology between adults and children, and a low incidence and severity of CARDS in children.

The search was then refined to include only CTs from the USA or UK, yielding 169 and 23, respectively (figure 1D); these countries have two of the largest and most highly regulated healthcare systems in the world, with comparable high expenditures on services and infrastructure.^50^

### Search refinement

CTs were manually screened and filtered to ensure that all eligible examples were identified. Studies were deemed relevant from their title, conditions treated and intervention(s) prescribed. Each CT was then thoroughly inspected to ensure that participants met the criteria either for ARDS alone (using the EACC criteria^16 28 51^ or Berlin definition criteria^18 28^) or for both ALI or ARDS and were interventional, so they involved administration of a pharmacological agent. The following exclusion criteria were applied to eliminate irrelevant CTs:

- Application of intervention for ARDS was not assessed or ARDS was not listed as a condition under investigation (29 USA and 3 UK).
- Participants did not meet the criteria for ALI or ARDS diagnosis (29 USA and 3 UK).
- Behavioural or mechanical interventions were assessed (28 USA and 4 UK).
- Paediatric participants were involved (11 USA).
- Healthy participants were involved (5 USA and 5 UK).
- Pregnant or recently postpartum participants were involved (5 USA).
- The investigational agent was used as a marker in an exploratory investigational trial (1 UK)

This refinement process identified 61 and 7 CTs from the USA and UK databases, respectively (figure 1E), before two CTs were eliminated due to duplication. This gave a total of 66 CTs for analysis (figure 1F), which are listed numerically by CT number (for ease of reference) in the Electronic Supporting Information.

### Patient and public involvement

No patients were involved in this study.

## Results

### Overview of the CT metrics data

**Figure 2:**
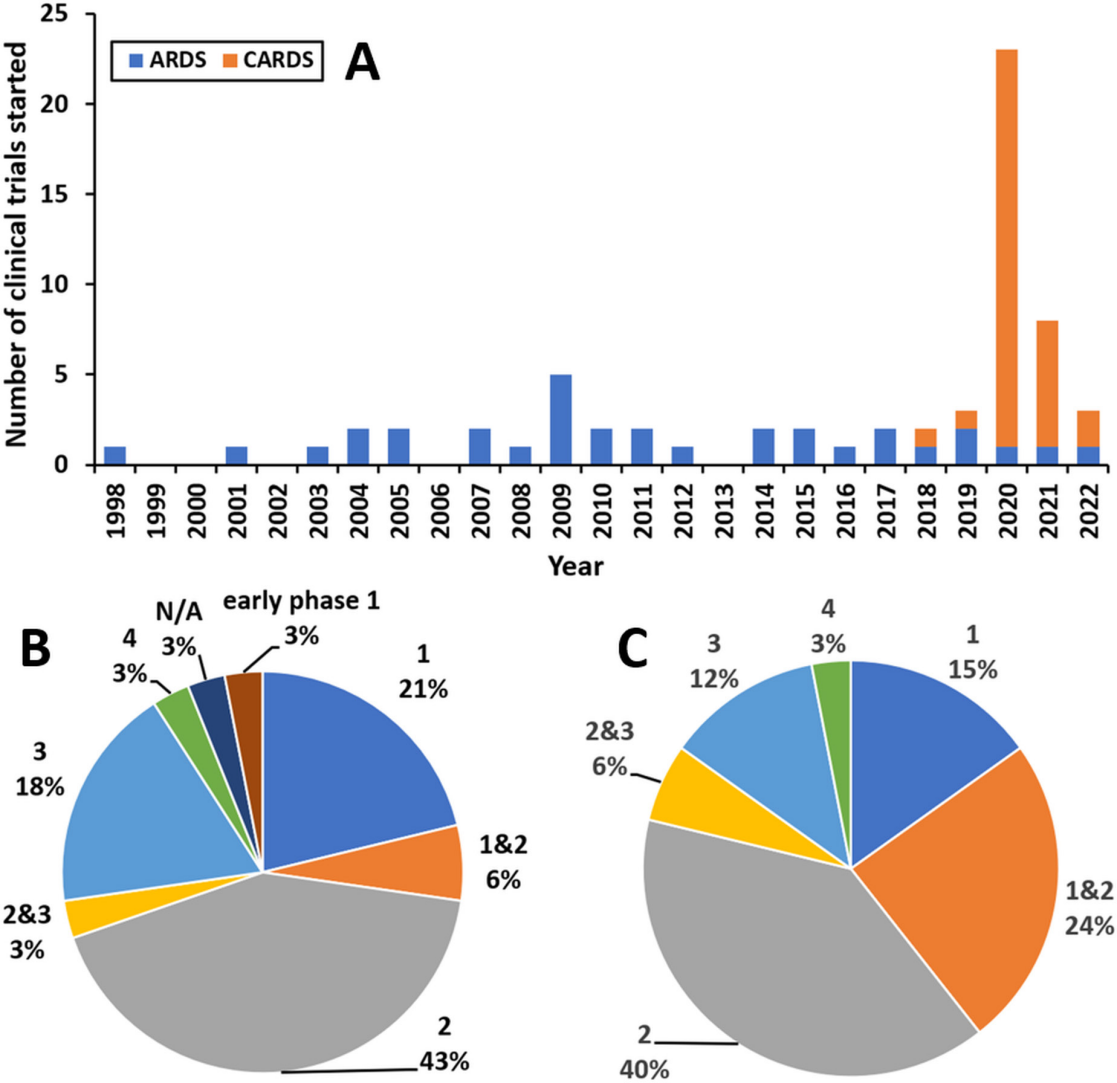
(A) Frequency of ARDS and CARDS CTs plotted by start year of study. (B) Stage of ARDS CTs in March 2022; N/A indicates trials where the stage was not specified. (C) Stage of CARDS CTs in March 2022; 0% of CARDS CTs were in early phase 1. ARDS, acute respiratory distress syndrome; CARDS, COVID-19 ARDS.

The CTs assessing new therapies for CARDS and ARDS were first categorised by the year in which they started (figure 2A). The number of ARDS CTs starting per year was typically 1–3, with a peak of 5 in 2009, yet the number stayed comparatively steady even throughout the pandemic. Strikingly, there was a significant increase in the number of CTs following the emergence of COVID-19. The number of CARDS CTs peaked at 22 starting in 2020, and then fell by more than half to 7 starting in 2021; the further drop in 2022 should be viewed with caution given that the present study took place in March 2022. Interestingly, over the whole period, there was the same number of CTs starting to investigate treatments for CARDS as for ARDS (n=33), and at the time of the study, 20 CTs had results published on ClinicalTrials.gov. One CARDS CT is shown as starting in 2018 (NCT03376854), which may at first seem anomalous, but the study shifted its focus from ARDS to CARDS as the COVID-19 pandemic transpired.

The phase of the CTs in March 2022 was next considered both for ARDS (figure 2B) and CARDS (figure 2C). As CARDS is a recent condition, there was a higher proportion of CARDS CTs in earlier phases than ARDS CTs, and fewer were completed with published results (n=6 vs 14, respectively). Many CTs were in their early stages: 41% measured adverse events of the interventions to assess safety in at least one of their primary outcomes, where an adverse event, in the context of a CT, is any untoward or unfavourable medical occurrence, including but not limited to an abnormal physical examination, abnormal laboratory finding, a symptom or disease, medication side effects, injury, psychological harm or trauma or death. Other primary and secondary outcomes assessed include 38% on mortality, 30% on MV duration, 9% on oxygenation, most commonly through the PaO_2_/FiO_2_ ratio, and 9% on a clinical biomarker specific to the intervention to assess its pharmacodynamic effect, for example, four CTs measured IL-6 or IL-8 concentrations as common inflammatory biomarkers.

**Figure 3:**
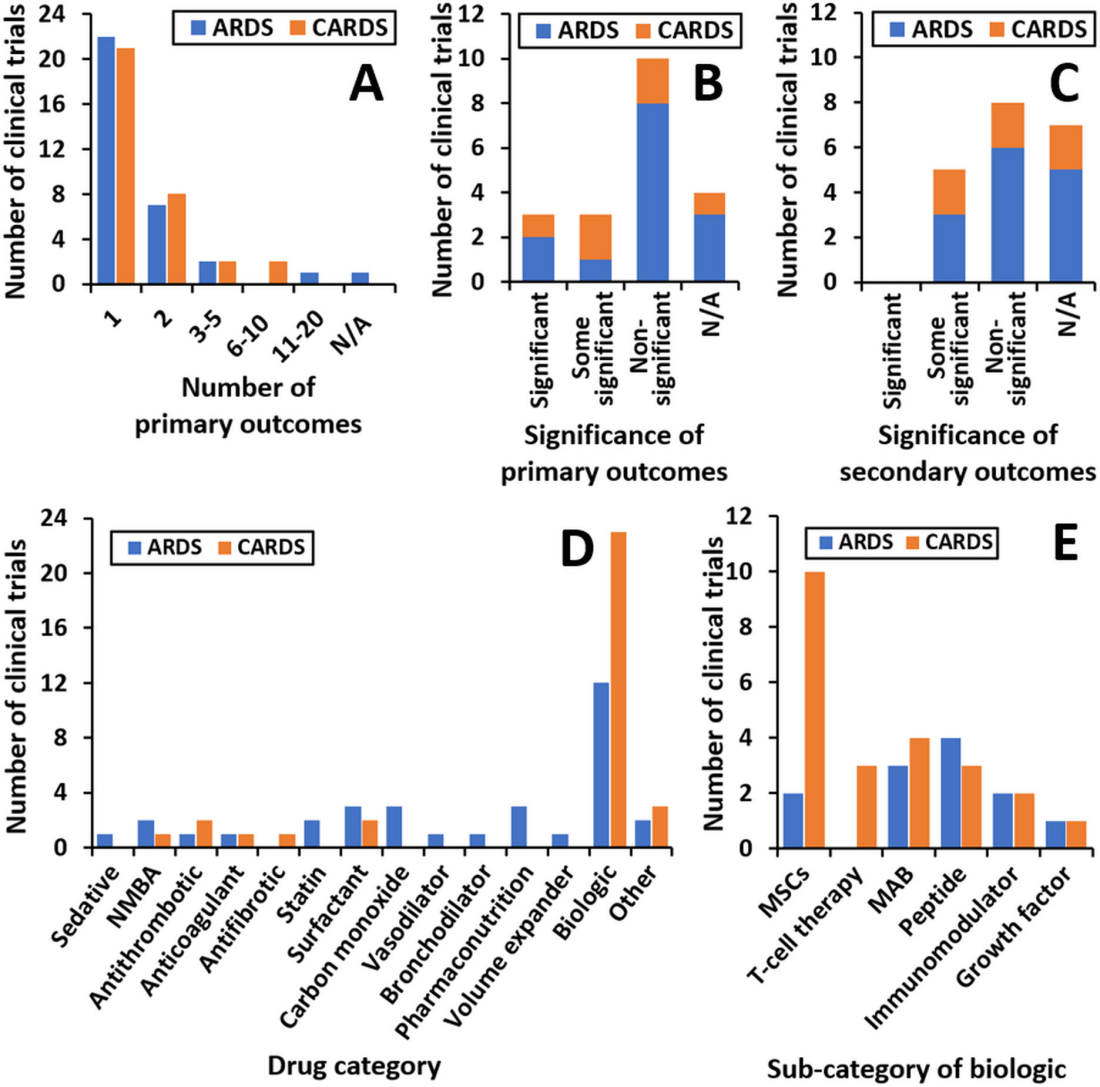
(A) The number of primary outcomes assessed in each CT. The number of CTs reporting a statistically significant (p<0.05) effect of the intervention on one or more of the (B) primary and (C) secondary outcomes; CTs categorised as ‘N/A’ when there were not enough data to determine whether the intervention significantly altered the primary or secondary outcomes. (D) Classes of drugs assessed and (E) biologics under investigation in the CTs for CARDS and ARDS. ARDS, acute respiratory distress syndrome; CARDS, COVID-19 ARDS; mAb, monoclonal antibody; NMBA, neuromuscular blocking agent.

Both ARDS and CARDS CTs assessed a range of clinical outcomes with the number of primary outcomes per CT shown (figure 3A). Of the CTs with primary outcomes, 65% had only one while 23% had two; commonly these CTs combined a primary outcome relating to safety with another relating to intervention efficacy. CARDS CTs included multiple primary outcomes, which is likely to be due to CT stages being combined due to the urgent need for new treatments with more CTs in phases 1 and 2 compared with those only in phase 1.

The significance of the primary (figure 3B) and secondary (figure 3C) outcomes was next considered for both ARDS and CARDS CTs. Of the CTs with published data, three CARDS and three ARDS CTs reported that the intervention tested significantly altered one or more of the primary outcomes assessed. This result translated to a higher percentage of CARDS CTs reporting significant results compared with ARDS CTs (50% vs 36%). None of them reported that the intervention significantly altered all of the secondary outcomes; however, three ARDS and two CARDS CTs reported that the intervention significantly altered one or more of them. This result may be explained by most CTs having had a higher number of secondary outcomes than primary outcomes, so it was less likely that an intervention would significantly affect all of them.

Given the wide variety of pathophysiologic pathways involved in ARDS, a diverse range of drug families were investigated as possible treatments (figure 3D). Antithrombotics, anticoagulants, NMBAs, surfactants and biologics were under investigation for ARDS and CARDS. Sedatives, statins, carbon monoxide (CO), bronchodilators, nutritional supplements and volume expanders were being tested for ARDS but not CARDS. An antifibrotic was under investigation for CARDS but not ARDS. The most common interventions in both ARDS and CARDS were biologics (figure 3E). In this case, monoclonal antibodies (mAbs), peptides, MSCs, immunomodulators and growth factors were assessed in both conditions, while T-cell therapy was assessed in CARDS only.

### Interventions demonstrating significant clinical benefit

A summary of the interventions exhibiting significant benefit to CARDS and/or ARDS patients is shown (table 2); some of the most interesting findings in terms of patient outcomes and/or clinical measures are noted, while a summary of interventions that exhibit unknown or no effect for both CARDS and ARDS patients can be found in the Electronic Supporting Information.

**Table 2:** A comparison of the effectiveness of therapeutic interventions in ARDS versus CARDS patients in CTs with published results

Intervention	ARDS	CARDS
Cytokine inhibitor (CERC-002)*	No trials → *Unknown effect*	**NCT04412057:** CERC-002 decreased mortality, respiratory failure and adverse events after day 28 → *improvement in patient outcomes*
C5 inhibitor (Ravulizumab)*	No trials → *Unknown effect*	**NCT04369469:** Ravulizumab dosing achieved complement inhibition → *improvement in clinical measures*
Antithrombotics*	**NCT00431379:** trial of Tenecteplase withdrawn → *Unknown effect*	**NCT04530604:** Defibrotide was deemed safe and tolerable, and no haemorrhagic or thrombotic complications were reported **NCT04357730:** Altepase improved oxygenation over baseline; mortality and the median number of VFD were reduced (not statistically significant) → *improvement in clinical measures*
Mesenchymal stem cells**	**NCT02804945:** data not yet available **NCT02097641:** MSC infusion led to higher mean scores for Acute Physiology and Chronic Health Evaluation III, minute ventilation and positive end-expiratory pressure, and decreased plasma concentrations of angiopoietin-2 → *improvement in clinical measures*	**NCT04345601, NCT04384445, NCT04629105, NCT04798716, NCT04905836:** data not yet available **NCT04490486:** trial withdrawn **NCT03042143:** no difference in any outcome measured **NCT04355728:** plasma levels of soluble TNF receptor 2 significantly increased; levels of TNFα and TNFβ significantly decreased **NCT04371393:** trial terminated **NCT04399889:** trial terminated → *improvement in clinical measures*
Anticoagulants***	**NCT00112164:** increased plasma protein C levels and decreased pulmonary dead space fraction, but no effect on VFD or 60-day mortality → *improvement in clinical measures and patient outcomes*	**NCT04397510:** nebulised heparin trial ongoing → *Unknown effect*
Carbon monoxide***	**NCT04870125, NCT03799874:** data not yet available **NCT02425579:** significantly increased carboxyhaemoglobin and reduced circulating mitochondrial DNA levels → *improvement in clinical measures*	No trials → *Unknown effect*
cAMP modification***	**NCT01274481:** nebulised iloprost significantly improved baseline PaO_2_ **NCT04417036:** inhaled pegylated adrenomedullin trial terminated **NCT00434993:** Albuterol did not affect mortality rates but did reduce VFD in a subset of patients → *improvement in clinical measures*	No trials → *Unknown effect*
Vasoactive intestinal peptide***	**NCT00004494:** modest decrease in plasma TNF-α levels was observed in most patients → *improvement in clinical measures*	**NCT04360096:** viptadil acetate, a synthetic vasoactive intestinal peptide analogue; trial terminated → *Unknown effect*
Renin-angiotensin system modification***	**NCT01597635:** GSK2586881 significantly reduced angiotensin II levels and increased angiotensin-(1-7), angiotensin-(1-5) and surfactant protein D levels → *improvement in clinical measures*	**NCT04778059:** USB002 trial terminated due to poor recruitment → *Unknown effect*

NCT numbers of the corresponding CT are listed where appropriate, and effects of the intervention in the two conditions are stated. The number of asteriks after each intervention indicates its effectiveness in treating the different conditions: effective in treating CARDS with unknown effectiveness in ARDS (marked *), effective in treating both conditions (marked **) and effective in treating ARDS with unknown effectiveness in CARDS (marked ***); where effectiveness is defined as improvements in patient outcomes and/or clinical measures.

ARDS, acute respiratory distress syndrome; cAMP, cyclic adenosine monophosphate; CARDS, COVID-19 ARDS; MSC, mesenchymal stem cell; TNF, tumour necrosis factor; VFD, ventilator free days.

The mAb CERC-002 (NCT04412057) inhibits lymphotoxin-like, inducible protein (LIGHT), a cytokine within the tumour necrosis factor (TNF) family, which induces the release of other inflammatory cytokines, including granulocyte-macrophage colony-stimulating factor.^38 40^ This CT reported a statistically significant increase in the number of subjects alive and free of respiratory failure at day 28 of CERC-002 treatment (83.9% vs 64.5%; p=0.0440).^38^

NCT02097641 assessed the administration of an allogeneic bone marrow-derived human MSC infusion to ARDS patients. The primary outcome determined that no infusion-associated adverse events occurred in either the drug arm (n=40) or placebo arm (n=20). Mortality was higher in the MSC infusion group (37.5% vs 25%), but the difference was not statistically significant. However, there was a statistically significant reduction in plasma angiopoietin-2 concentration, a biomarker of pulmonary vascular injury, in the MSC infusion group. Study limitations affected the depth of conclusions, despite improvements to oxygenation seen in the MSC infusion group.^52^ NCT04355728 assessed the safety and efficacy of an infusion-containing human-umbilical cord-derived MSCs to COVID-19-induced ALI/ARDS patients. The incidence of adverse events was significantly reduced in the treatment group (2 vs 8 events; p=0.04), but no other outcomes were significantly improved.

NCT01597635 investigated the safety of GSK2586881, a recombinant human angiotensin-converting enzyme-2 (ACE2) peptide in ALI or ARDS, to modify the renin–angiotensin system (RAS) and alleviate disease pathology. Primary outcome measure analysis revealed that the peptide was safe, with no significant difference in adverse events in the treatment versus control group, although the basal rate of adverse events remained high. The pharmacokinetic and pharmacodynamic objectives were met in a planned futility analysis (n=39). The study was not powered to detect changes in clinical outcomes or acute physiology; however, a non-significant decrease in plasma IL-6 concentration compared with the placebo was observed.^53^

Treatment with an alteplase bolus (NCT04357730) led to statistically significant improvements in PaO_2_/FiO_2_ ratios, a measure of oxygenation compared with untreated controls. At 24 hours, controls (n=17) exhibited a median PaO_2_/FiO_2_ ratio of 146.7, compared with the median ratio of 144 in the intervention group (n=19; p>0.05), hence showing higher oxygenation levels. However, at 48 hours, the median values were 125 and 157.1, respectively, which translated into a statistically significant improvement when compared over 168 hours from baseline (p<0.017).^54^A PaO_2_/FiO_2_ ≥200 or a 50% increase in the ratio was observed in two control participants (11.8%) and nine interventional participants (47.4%), indicating a therapeutic effect of the treatment (p=0.03).^54^

## Discussion

### Therapeutics effective in treating CARDS that have an unknown effect in treating ARDS

The present systematic review has identified a range of therapies assessed for their effectiveness in both ARDS and CARDS, and it has highlighted several treatments that at the time of the study in March 2022 showed therapeutic efficacy in CARDS with potential in the treatment of ARDS (table 2, interventions marked *). The marked expansion in testing of anti-inflammatory biologics for the treatment of both conditions is likely because inflammation has been identified as part of the detrimental pathology of CARDS.^38 40 42^ This link provided, in part, the motivation for us to review which treatments developed for CARDS, following the onset of the pandemic, also show potential in the treatment of ARDS, when developed from other illnesses.

A mAb was inferred to have favourable effects in CARDS that may be applicable to ARDS. CERC-002 significantly increased the number of CARDS patients free of respiratory failure by day 28 (NCT04412057).^40^ CERC-002 inhibits LIGHT, which drives inflammation, T-cell proliferation and cytokine release, contributing to tissue damage and fibrosis.^38 40^ LIGHT levels are raised in COVID-19 patients and serum concentration is proportional to infection severity, hence its inhibition may mitigate propagation of the cytokine storm and CARDS progression. The addition of CERC-002 to ARDS standard care may also improve patient outcomes,^38^ particularly in conditions such as bacterial sepsis, where LIGHT levels are raised.^55^

C5 is a major component of the complement cascade and is positively associated with COVID-19 infection severity.^56^ C5a has additional prothrombotic actions and upregulates neutrophil extracellular trap (NET) formation, which directly damages alveolar and epithelial cells, inducing cell death.^12 57^ Ravulizumab, a mAb trialled in COVID-19 ALI/ARDS achieved terminal inhibition of the complement system, which was thought to prevent disease progression (NCT04369469).^56^ However, this CT was terminated for futility, since this did not translate into positive clinical outcomes and instead increased mortality. When considering the hyperimmune response during a COVID-19 infection, however, and the variable nature of ARDS, timing of complement inhibition is crucial. Complement activation may be beneficial in the first week of the disease but have devastating outcomes as the condition progresses. Thus, Ravulizumab may be a successful therapy only in the second or third weeks of CARDS or ARDS.^58^

Despite the association of CARDS with a pro-coagulant phenotype, and higher thromboembolic disease manifestations than ARDS,^23 31^ thromboembolic complications remain a common cause of death in ARDS patients.^22^ Tissue plasminogen activators such as alteplase (NCT04357730) and tenecteplase (NCT00431379) may be more efficacious than traditional thrombolytic therapies, as they exhibit higher specificity yet have a comparable bleeding risk. By activating plasmin, these agents degrade fibrin, which accumulates and deposits in the pulmonary vasculature in a similar way in both CARDS and ARDS, leading to fibrosis and clot formation.^31 59^ Alteplase administration significantly improved PaO_2_/FiO_2_ ratios in CARDS patients by dissolving thrombi to increase blood flow (NCT04357730), also inferring potential efficacy in ARDS.^54^

### Therapeutics effective in treating both CARDS and ARDS

The present review has also identified several therapies that show efficacy in both ARDS and CARDS (table 2, interventions marked **). MSCs are being investigated in both ARDS (NCT02804945, NCT02097641) and CARDS (NCT04345601, NCT04629105, NCT04490486, NCT04384445, NCT04355728, NCT04371393, NCT04798716, NCT04399889, NCT04905836, NCT03042143) and have shown therapeutic potential by facilitating greater increases in oxygenation following higher viability infusions (NCT02097641, NCT04355728). These trials indicate that MSC infusions can lead to a reduction in inflammatory and immunological responses following trauma such as VILI, resulting in improved lung function.^44^ Although immunomodulatory effects of MSCs may be particularly applicable to CARDS, by inhibiting T-cell production of proinflammatory cytokines,^29 44^ anti-inflammatory effects may suppress ARDS progression. Additionally, CARDS research may lead to advances in MSC isolation and culture to overcome current limitations around viability, purity and specificity.^44 52^

### Therapeutics effective in treating ARDS that have an unknown effect in treating CARDS

Lastly, the present review has identified agents that may show benefit in ARDS but at the time of the study in March 2022 had not resolved and known effect in CARDS (table 2, interventions marked ***). These were anticoagulants (NCT00112164, NCT04397510), CO (NCT04870125, NCT02425579, NCT03799874), cyclic adenosine monophosphate (cAMP) level modification factors (NCT00434993, NCT01274481, NCT04417036), vasoactive intestinal peptide (NCT00004494, NCT04360096) and RAS modification (NCT01597635, NCT04778059).

Besides their principal roles as anticoagulants, activated protein C (NCT00112164) and heparin (NCT04397510) may have anti-inflammatory actions that improve alveolar-capillary injury. Preclinical studies with heparin have demonstrated anti-inflammatory effects, but to date, these have not translated into improved patient outcomes. There is speculation that heparin may decrease cytokine levels implicated in cytokine storm in CARDS.^25 59^ Such effects would likely result in an overall improvement in patient outcomes.

CO has vasoactive, antithrombotic and anti-inflammatory actions and can decrease levels of proinflammatory cytokines and increase anti-inflammatory cytokines. Experimental data suggest that CO may contribute to the resolution of pulmonary inflammation, and it may be particularly effective in ARDS that coexists with sepsis or organ dysfunction.^60^ Despite being shown to be safely administered in controlled research-based environments, its safety aspects remain a concern given the widely known toxicity of this gas (NCT04870125, NCT03799874, NCT02425579).^60^

Adrenomedullin raises intracellular cAMP levels in endothelial cells to maintain endothelial barrier integrity, so it may protect vascular function and attenuate the increase in pulmonary permeability following inflammation in conditions like ARDS.^29 61^ Indeed, cAMP-level modification factors show therapeutic effects in ARDS, although their side effects may limit their clinical use.^62^ Upregulation of plasma cAMP levels may be a novel approach to treat COVID-19 infection, potentially attenuating development of the condition.^63^ cAMP modification has been tested in ARDS trials with the most notable improvement in clinical measures coming from significantly improved baseline PaO_2_ through the use of nebulised iloprost (NCT01274481).

Vasoactive intestinal peptide is also under investigation in ARDS (NCT00004494) and CARDS (NCT04360096). The peptide is speculated to protect alveolar type II cells from inflammatory damage, preserve surfactant production and have immunomodulatory effects, such as inhibiting nuclear factor-kappa B signalling. These effects reduce production of TNF-α, interleukins and other proinflammatory cytokines, averting the cytokine storm in CARDS; these actions may also reduce or alleviate inflammation in ARDS.^10 43 46^

The RAS is central to pulmonary vascular tone regulation.^64 65^ ACE-2 cleaves angiotensin-II (Ang-II) to produce Ang1-7.^29 40 47 64 65^ These two products have opposing roles with Ang-II imposing vasoconstriction and Ang1-7 imposing vasodilation.^29 64 65^ Ang-II levels are detectably higher in ALI or ARDS (NCT01597635), thus Ang-II is believed to promote lung injury. GSK2586881, a recombinant human ACE-2 administered to ARDS patients replicated the anticipated clinical effects at the planned futility analysis (NCT01597635). GSK2586881 infusions produced a dramatic and persistent reduction in Ang-II, accompanied by a persistent elevation in Ang1-7 levels, which correlated with GSK2586681 plasma concentrations. While primary safety, and pharmacokinetic and pharmacodynamic objectives were met, a greater understanding of the role of RAS in ARDS pathophysiology is also needed (NCT01597635).^47^ This may be achieved by administering USB002, a pharmaceutically formulated form of Ang1-7, in CARDS (NCT04778059). However, the primary objective of this trial was to assess the safety of USB002 and the trial was terminated prematurely. Further, RAS may have differing roles in ARDS and CARDS, since the SARS-CoV-2 virus renders ACE-2 ineffective on binding (NCT04778059).^64^

Although several biologics were identified that did not exhibit any beneficial effects in either condition (NCT02622724, NCT00201409, NCT04616586, NCT04402060, NCT04351243), this result could in part be explained by the need for a more personalised treatment approach,^31 40–42^ given the widely variable nature of ARDS pathogenesis. Stratification of patients into study groups with more defined symptoms, biomarkers and/or pathological features may help to prevent the ineffective use of biologics and aid success rates, particularly where the therapeutic target is, for example, a specific inflammatory marker.^40 42^

## Conclusions

In the present work, through a systematic review of freely available data from CTs, we have highlighted a number of novel and promising interventions for the treatment of CARDS as well as ARDS developed from illnesses other than COVID-19. Given their distinct but overlapping pathologies, some interventions show promise in being more efficacious in CARDS patients, such as antithrombotics and cytokine inhibitors. Personalised treatment through the increased use of biologics was identified as a promising focus of future research for ARDS as well as CARDS patients, as were complement system modifiers. Interventions such as cell-based therapies, on the other hand, have already been shown to be efficacious in both conditions, which is attributed to their broad mechanism of action and the overlap in clinical aspects of their pathologies. We also recognise that, to date, the most successful interventions for CARDS have been antiviral drugs such as remdesivir,^66^ Paxlovid (nirmatrelvir and ritonavir^67^) and molnupiravir^68^; however, these are unlikely to be of benefit to ARDS patients who do not have a viral infection, and CTs assessing these compounds were not identified in our searches. As ongoing CTs continue, growth of the current knowledge base will lead to a wider range of clinically approved therapies for CARDS, which, in turn, is likely to influence the future management of ARDS. Refinements in both the design of trials and selection of patient groups may facilitate trial success, and the research will surely lead to a greater understanding of the pathophysiology and optimal treatment strategy for both CARDS and ARDS.

10.1136/bmjresp-2022-001525.supp1 Supplementary data

## Data Availability

Data are available in a public, open access repository.

## References

[1] Summers C, Singh NR, Worpole L, et al. Incidence and recognition of acute respiratory distress syndrome in a UK intensive care unit. Thorax 2016;71:1050–1. 10.1136/thoraxjnl-2016-208402 27552782 PMC5099179

[2] Davydow DS, Desai SV, Needham DM, et al. Psychiatric morbidity in survivors of the acute respiratory distress syndrome: a systematic review. Psychosom Med 2008;70:512–9. 10.1097/PSY.0b013e31816aa0dd 18434495 PMC10885706

[3] Reynolds H, McCunn M, Borg U, et al. Acute respiratory distress syndrome: estimated incidence and mortality rate in a 5 million-person population base. Crit Care 1998;2:29–34. 10.1186/cc121 11056707 PMC28999

[4] Ervin JN, Rentes VC, Dibble ER, et al. Evidence-based practices for acute respiratory failure and acute respiratory distress syndrome: a systematic review of reviews. Chest 2020;158:2381–93. 10.1016/j.chest.2020.06.080 32682771 PMC7768938

[5] Terzi E, Zarogoulidis K, Kougioumtzi I, et al. Acute respiratory distress syndrome and pneumothorax. J Thorac Dis 2014;6:S435–42. 10.3978/j.issn.2072-1439.2014.08.34 25337400 PMC4203978

[6] Williams AE, Chambers RC. The mercurial nature of neutrophils: still an enigma in ARDS Am J Physiol Lung Cell Mol Physiol 2014;306:L217–30. 10.1152/ajplung.00311.2013 24318116 PMC3920201

[7] Cardinal-Fernandez P, Ballen Barragan A, Lorente J. ARDS: A Clinical Syndrome or A Pathological Entity? Annual Update in Intensive Care and Emergency Medicine 2014, 1st ed. Cham: Springer, 2014: 219–27.

[8] Coudroy R, Boissier F, Thille A. Acute Respiratory Distress Syndrome (ARDS): Definition, Incidence and Outcome. Acute Respiratory Distress Syndrome, 1st ed. Cham: Springer, 2017: 1–14.

[9] Silva P, Rocco P. Pathophysiology of Acute Respiratory Distress Syndrome. Acute Respiratory Distress Syndrome, 1st ed. Cham: Springer, 2017: 15–23.

[10] Pierrakos C, Karanikolas M, Scolletta S, et al. Acute respiratory distress syndrome: pathophysiology and therapeutic options. J Clin Med Res 2012;4:7–16. 10.4021/jocmr761w 22383921 PMC3279495

[11] Matthay MA, Zimmerman GA. Acute lung injury and the acute respiratory distress syndrome. Am J Respir Cell Mol Biol 2005;33:319–27. 10.1165/rcmb.F305 16172252 PMC2715340

[12] Matthay MA, Zemans RL, Zimmerman GA, et al. Acute respiratory distress syndrome. Nat Rev Dis Primers 2019;5:18. 10.1038/s41572-019-0069-0 30872586 PMC6709677

[13] Davies A, Moore C. The respiratory system: basic science and clinical conditions, 2nd ed. Edinburgh: Churchill Livingstone, 2014: 11–69.

[14] Lawrence H, Moore T. Crash course respiratory medicine, 5th ed. London: Elsevier Limited, 2019: 3–70.

[15] Han S, Mallampalli RK. The role of surfactant in lung disease and host defense against pulmonary infections. Ann Am Thorac Soc 2015;12:765–74. 10.1513/AnnalsATS.201411-507FR 25742123 PMC4418337

[16] Taylor Thompson B, Chambers RC, Liu KD, et al. Acute respiratory distress syndrome. N Engl J Med 2017;377:562–72. 10.1056/NEJMra1608077 28792873

[17] Marshall R, Bellingan G, Laurent G. The acute respiratory distress syndrome: fibrosis in the fast lane. Thorax 1998;53:815–7. 10.1136/thx.53.10.815 10193364 PMC1745098

[18] Lewis SR, Pritchard MW, Thomas CM, et al. Pharmacological agents for adults with acute respiratory distress syndrome. Cochrane Database Syst Rev 2019. 10.1002/14651858.CD004477.pub3 PMC6646953 31334568

[19] Collins SR, Blank RS. Approaches to refractory hypoxemia in acute respiratory distress syndrome: current understanding, evidence, and debate. Respir Care 2011;56:1573–82. 10.4187/respcare.01366 22008398

[20] DiSilvio B, Young M, Gordon A, et al. Complications and outcomes of acute respiratory distress syndrome. Crit Care Nurs Q 2019;42:349–61. 10.1097/CNQ.0000000000000275 31449145

[21] Slutsky AS, Ranieri VM. Ventilator-induced lung injury. N Engl J Med 2013;369:2126–36. 10.1056/NEJMra1208707 24283226

[22] Lew TWK, Kwek T-K, Tai D, et al. Acute respiratory distress syndrome in critically ill patients with severe acute respiratory syndrome. JAMA 2003;290:374–80. 10.1001/jama.290.3.374 12865379

[23] Papazian L, Forel J-M, Gacouin A, et al. Neuromuscular blockers in early acute respiratory distress syndrome. N Engl J Med 2010;363:1107–16. 10.1056/NEJMoa1005372 20843245

[24] Nasrullah A, Virk S, Shah A, et al. Acute respiratory distress syndrome and the use of inhaled pulmonary vasodilators in the COVID-19 era: a narrative review. Life (Basel) 2022;12:1766. 10.3390/life12111766 36362921 PMC9695622

[25] Matthay MA, Brower RG, Carson S, et al. Randomized, placebo-controlled clinical trial of an aerosolized Β₂-Agonist for treatment of acute lung injury. Am J Respir Crit Care Med 2011;184:561–8. 10.1164/rccm.201012-2090OC 21562125 PMC3175548

[26] Gao Smith F, Perkins GD, Gates S, et al. Effect of intravenous Β-2 agonist treatment on clinical outcomes in acute respiratory distress syndrome (BALTI-2): a multicentre, randomised controlled trial. Lancet 2012;379:229–35. 10.1016/S0140-6736(11)61623-1 22166903 PMC3266479

[27] Perkins GD, Gates S, Park D, et al. The beta agonist lung injury trial prevention. A randomized controlled trial. Am J Respir Crit Care Med 2014;189:674–83. 10.1164/rccm.201308-1549OC 24392848 PMC3983838

[28] Hyzy RC, McSparron J. Evidence-based critical care. In: Hyzy R, McSparron J, eds. Management of Acute Respiratory Distress Syndrome, 2nd ed. Cham: Springer, 2020: 162–7. 10.1007/978-3-030-26710-0

[29] Curley GF, Laffey JG. Future therapies for ARDS. Intensive Care Med 2015;41:322–6. 10.1007/s00134-014-3578-z 25472571 PMC7079876

[30] Holliday ZM, Alnijoumi MM, Reed MA, et al. Neutrophils and secondary infections in COVID-19 induced acute respiratory distress syndrome. New Microbes New Infect 2021;44:100944. 10.1016/j.nmni.2021.100944 34567574 PMC8452528

[31] Griffiths M, Meade S, Summers C, et al. RAND appropriateness panel to determine the applicability of UK guidelines on the management of acute respiratory distress syndrome (ARDS) and other strategies in the context of the COVID-19 pandemic. Thorax 2022;77:129–35. 10.1136/thoraxjnl-2021-216904 34045363

[32] Aslan A, Aslan C, Zolbanin NM, et al. Acute respiratory distress syndrome in COVID-19: possible mechanisms and therapeutic management. Pneumonia (Nathan) 2021;13:14. 10.1186/s41479-021-00092-9 34872623 PMC8647516

[33] Ferrando C, Suarez-Sipmann F, Mellado-Artigas R, et al. Correction to: clinical features, ventilatory management, and outcome of ARDS caused by COVID-19 are similar to other causes of ARDS. Intensive Care Med 2021;47:144–6. 10.1007/s00134-020-06251-8 33263817 PMC7709481

[34] Attaway AH, Scheraga RG, Bhimraj A, et al. Severe COVID-19 pneumonia: pathogenesis and clinical management. BMJ 2021;372:n436. 10.1136/bmj.n436 33692022

[35] Gattinoni L, Marini JJ. Isn’t it time to abandon ARDS? The COVID-19 lesson. Crit Care 2021;25:326. 10.1186/s13054-021-03748-6 34488807 PMC8419818

[36] Haudebourg A-F, Perier F, Tuffet S, et al. Respiratory mechanics of COVID-19– versus non–COVID-19–associated acute respiratory distress syndrome. Am J Respir Crit Care Med 2020;202:287–90. 10.1164/rccm.202004-1226LE 32479162 PMC7365370

[37] Bain W, Yang H, Shah FA, et al. COVID-19 versus non–COVID-19 acute respiratory distress syndrome: comparison of demographics, physiologic parameters, inflammatory biomarkers, and clinical outcomes. Ann Am Thorac Soc 2021;18:1202–10. 10.1513/AnnalsATS.202008-1026OC 33544045 PMC8328355

[38] Perlin DS, Neil GA, Anderson C, et al. Randomized, double-blind, controlled trial of human anti-LIGHT monoclonal antibody in COVID-19 acute respiratory distress syndrome. J Clin Invest 2022;132:e153173. 10.1172/JCI153173 34871182 PMC8803341

[39] Gibson PG, Qin L, Puah SH. COVID-19 acute respiratory distress syndrome (ARDS): clinical features and differences from typical pre-COVID-19 ARDS. Med J Aust 2020;213:54–56. 10.5694/mja2.50674 32572965 PMC7361309

[40] Perlin DS, Neil GA, Anderson C, et al. CERC-002, a human anti-LIGHT MAB reduces respiratory failure and death in hospitalized COVID-19 ARDS patients. Pharmacol Ther [Preprint] 2021. 10.1101/2021.04.03.21254748

[41] Xia B, Shen X, He Y, et al. SARS-CoV-2 envelope protein causes acute respiratory distress syndrome (ARDS)-Like pathological damages and constitutes an antiviral target. Cell Res 2021;31:847–60. 10.1038/s41422-021-00519-4 34112954 PMC8190750

[42] Lee KMC, Achuthan AA, Hamilton JA. GM-CSF: a promising target in inflammation and autoimmunity. Immunotargets Ther 2020;9:225–40. 10.2147/ITT.S262566 33150139 PMC7605919

[43] Javitt JC. Perspective: the potential role of vasoactive intestinal peptide in treating COVID-19. Preprints [Preprint] 2020. 10.22541/au.158940764.42332418

[44] Zanirati G, Provenzi L, Libermann LL, et al. Stem cell-based therapy for COVID-19 and ARDS: a systematic review. Npj Regen Med 2021;6. 10.1038/s41536-021-00181-9 PMC8575895 34750382

[45] Dharra R, Kumar Sharma A, Datta S. Emerging aspects of cytokine storm in COVID-19: the role of proinflammatory cytokines and therapeutic prospects. Cytokine 2023;169:156287. 10.1016/j.cyto.2023.156287 37402337 PMC10291296

[46] Youssef JG, Said S, Youssef G, et al. Treatment of acute respiratory distress syndrome with vasoactive intestinal peptide. Medicine & Pharmacology [Preprint] 2020. 10.20944/preprints202007.0453.v1

[47] Zhang J, Huang X, Ding D, et al. Comparative study of acute lung injury in COVID-19 and non-COVID-19 patients. Front Med 2021;8:666629. 10.3389/fmed.2021.666629 PMC8415545 34485324

[48] Hendrickson KW, Peltan ID, Brown SM. The epidemiology of acute respiratory distress syndrome before and after Coronavirus disease 2019. Crit Care Clin 2021;37:703–16. 10.1016/j.ccc.2021.05.001 34548129 PMC8449138

[49] Pu D, Zhai X, Zhou Y, et al. A narrative review of COVID-19-related acute respiratory distress syndrome (CARDS): 'typical' or 'atypical' ARDS. Ann Transl Med 2022;10:908. 10.21037/atm-22-3717 36111011 PMC9469157

[50] Fradinho JMS, Nightingale DJ, Fradinho MTW. Systems-of-systems perspective on healthcare: insights from two multi-method exploratory cases of leading U.S. and U.K. hospitals. IEEE Systems Journal 2014;8:795–802. 10.1109/JSYST.2013.2260091

[51] Bernard GR, Artigas A, Brigham KL, et al. Report of the American-European consensus conference on ARDS: definitions, mechanisms, relevant outcomes and clinical trial coordination. Intensive Care Med 1994;20:225–32. 10.1007/BF01704707 8014293

[52] Matthay MA, Calfee CS, Zhuo H, et al. Treatment with allogeneic mesenchymal stromal cells for moderate to severe acute respiratory distress syndrome (START study): a randomised phase 2A safety trial. Lancet Respir Med 2019;7:154–62. 10.1016/S2213-2600(18)30418-1 30455077 PMC7597675

[53] Khan A, Benthin C, Zeno B, et al. A pilot clinical trial of recombinant human angiotensin-converting enzyme 2 in acute respiratory distress syndrome. Crit Care 2017;21:234. 10.1186/s13054-017-1823-x 28877748 PMC5588692

[54] Barrett CD, Moore HB, Moore EE, et al. Study of Alteplase for respiratory failure in SARS-CoV-2 COVID-19. Chest 2022;161:710–27. 10.1016/j.chest.2021.09.024 34592318 PMC8474873

[55] Qu H-Q, Snyder J, Connolly J, et al. Circulating LIGHT (TNFSF14) and Interleukin-18 levels in sepsis-induced multi-organ injuries. Biomedicines 2022;10:264. 10.3390/biomedicines10020264 35203474 PMC8869623

[56] McEneny-King AC, Monteleone JPR, Kazani SD, et al. Pharmacokinetic and pharmacodynamic evaluation of ravulizumab in adults with severe coronavirus disease 2019. Infect Dis Ther 2021;10:1045–54. 10.1007/s40121-021-00425-7 33826106 PMC8024938

[57] Cesta MC, Zippoli M, Marsiglia C, et al. The role of Interleukin-8 in lung inflammation and injury: implications for the management of COVID-19 and hyperinflammatory acute respiratory distress syndrome. Front Pharmacol 2021;12:808797. 10.3389/fphar.2021.808797 35095519 PMC8790527

[58] Java A, Apicelli AJ, Liszewski MK, et al. The complement system in COVID-19: friend and foe? JCI Insight 2020;5:e140711. 10.1172/jci.insight.140711 32554923 PMC7455060

[59] Sebag SC, Bastarache JA, Ware LB. Therapeutic modulation of coagulation and fibrinolysis in acute lung injury and the acute respiratory distress syndrome. Curr Pharm Biotechnol 2011;12:1481–96. 10.2174/138920111798281171 21401517 PMC3893117

[60] Goebel U, Wollborn J. Carbon Monoxide in intensive care medicine—time to start the therapeutic application Intensive Care Med Exp 2020;8:2. 10.1186/s40635-020-0292-8 31919605 PMC6952485

[61] Kubo K, Tokashiki M, Kuwasako K, et al. Biological properties of Adrenomedullin conjugated with polyethylene glycol. Peptides 2014;57:118–21. 10.1016/j.peptides.2014.05.005 24874704

[62] Wu R, Lin S-Y, Zhao H-M. Albuterol in the treatment of acute respiratory distress syndrome: a meta-analysis of randomized controlled trials. World J Emerg Med 2015;6:165–71. 10.5847/wjem.j.1920-8642.2015.03.001 26401175 PMC4566004

[63] Chiang C-C, Korinek M, Cheng W-J, et al. Targeting neutrophils to treat acute respiratory distress syndrome in Coronavirus disease. Front Pharmacol 2020;11:572009. 10.3389/fphar.2020.572009 33162887 PMC7583590

[64] Cure E, Cumhur Cure M, Vatansev H. Central involvement of SARS-CoV-2 may aggravate ARDS and hypertension. J Renin Angiotensin Aldosterone Syst 2020;21:1470320320972015. 10.1177/1470320320972015 33169637 PMC7658518

[65] Kleinsasser A, Pircher I, Treml B, et al. Recombinant angiotensin-converting enzyme 2 suppresses pulmonary vasoconstriction in acute hypoxia. Wilderness Environ Med 2012;23:24–30. 10.1016/j.wem.2011.09.002 22441085

[66] Lee TC, Murthy S, Del Corpo O, et al. Remdesivir for the treatment of COVID-19: a systematic review and meta-analysis. Clin Microbiol Infect 2022;28:1203–10. 10.1016/j.cmi.2022.04.018 35598856 PMC9117160

[67] Najjar-Debbiny R, Gronich N, Weber G, et al. Effectiveness of Paxlovid in reducing severe Coronavirus disease 2019 and mortality in high-risk patients. Clin Infect Dis 2023;76:e342–9. 10.1093/cid/ciac443 35653428 PMC9214014

[68] Benaicha K, Khenhrani RR, Veer M, et al. Efficacy of Molnupiravir for the treatment of mild or moderate COVID-19 in adults: a meta-analysis. Cureus 2023;15:e38586. 10.7759/cureus.38586 37284377 PMC10239651

