# Supplementary material for: Acute respiratory distress syndrome: potential of therapeutic interventions effective in treating progression from COVID-19 to treat progression from other illnesses—a systematic review
Source: BMJ Open Respir Res. 2023 Sep 1;10(1):e001525. doi: 10.1136/bmjresp-2022-001525 (PMC10476125; doi:10.1136/bmjresp-2022-001525)
Supplement: Supplementary data [file bmjresp-2022-001525supp001.pdf]

Acute respiratory distress syndrome: potential of therapeutic interventions effective in treating progression from COVID-19 to treat progression from other illnesses – a systematic review

Emma J. Ragel[1], Lynda K. Harris[1,2,3,4], Richard A. Campbell[1*]

[1]Division of Pharmacy and Optometry, Faculty of Biology, Medicine and Health, The University of Manchester, Manchester, M13 9PL, United Kingdom

[2]Maternal and Fetal Health Research Centre, Division of Developmental Biology and Medicine, Faculty of Biology, Medicine and Health, The University of Manchester, Oxford Road, Manchester, M13 9WL, United Kingdom

[3]St Mary's Hospital, Manchester University NHS Foundation Trust, Manchester Academic Health Science Centre, Manchester, M13 9WL, United Kingdom

[4]Olson Center for Women's Health, University of Nebraska Medical Center, Omaha, NE 68198, United States

* to whom correspondence should be addressed: richard.campbell@manchester.ac.uk

Electronic Supporting Information

NCT number	Title	Primary outcome(s)	Start year	Link
NCT00004494	Phase I Study of Vasoactive Intestinal Peptide in Patients With Acute	N/A	1998	https://www.clinicaltrials.gov/ct2/show/NCT00004494?term=drug&type=Intr&cond=%22acute+respiratory+distr

	Respiratory Distress Syndrome and Sepsis			ess+syndrome%22&cntry=US&age=1&draw=3&rank=73
NCT00112164	Activated Protein C to Treat Acute Lung Injuries	1. Number of ventilator-free days (measured at Day 28)	2005	https://www.clinicaltrials.gov/ct2/show/NCT00112164?term=drug&type=Intr&cond=%22acute+respiratory+distress+syndrome%22&cntry=US&age=1&draw=3&rank=97
NCT00201409	A Randomized Trial of GM-CSF in Patients With ALI/ARDS	1. Ventilator-free Days During Days 1-28 [Time Frame: Measured at Day 28]	2004	https://www.clinicaltrials.gov/ct2/show/NCT00201409?term=drug&type=Intr&cond=%22acute+respiratory+distress+syndrome%22&cntry=US&age=1&draw=4&rank=44
NCT00215553	KL_4 Surfactant Treatment in Patients With ARDS	1. Incidence of Patients Being Alive and Not Receiving Mechanical Ventilation for ≥48 h at the End of Day 28. [Time Frame: Through 28 days]	2001	https://www.clinicaltrials.gov/ct2/show/NCT00215553?term=drug&type=Intr&cond=%22acute+respiratory+distress+syndrome%22&cntry=US&age=1&draw=3&rank=98
NCT00233207	IC14 Antibodies to Treat Individuals With Acute Lung Injury	1. Alveolar lavage concentrations of interleukin-8 (measured post-treatment at Days 2, 3, 6, 7, and 8)	2005	https://www.clinicaltrials.gov/ct2/show/NCT00233207?term=drug&type=Intr&cond=%22acute+respiratory+distress+syndrome%22&cntry=US&age=1&draw=3&rank=79
NCT00431379	Treatment of acute respiratory distress syndrome with tenecteplase: a dose escalation pilot study	1. Survival to Discharge<br>2. Safety Analysis of Bleeding Complications	2007	https://www.clinicaltrials.gov/ct2/show/NCT00431379?term=drug&type=Intr&cond=%22acute+respiratory+distress+syndrome%22&cntry=US&age=1&draw=4&rank=12

NCT00434993	Drug study of albuterol to treat acute lung injury (ALTA)	1. Number of Ventilator Free Days (VFD) [Time Frame: Determined 28 days after a subject entered the study]	2007	https://www.clinicaltrials.gov/ct2/show/NCT00434993?term=drug&type=Intr&cond=%22acute+respiratory+distress+syndrome%22&cntry=US&age=1&draw=4&rank=29
NCT00682500	Calfactant for Direct Acute Respiratory Distress Syndrome (CARDS)	1. Mortality Rate [Time Frame: 90 days]	2008	https://www.clinicaltrials.gov/ct2/show/NCT00682500?term=drug&type=Intr&cond=%22acute+respiratory+distress+syndrome%22&cntry=US&age=1&draw=3&rank=81
NCT00742482	Efficacy and Safety of 3 Doses of HL10 Given at Fixed Time Intervals Compared to Standard Therapy	1. 28 days mortality [Time Frame: 28 days]	2003	https://www.clinicaltrials.gov/ct2/show/NCT00742482?term=drug&type=Intr&cond=%22Acute+respiratory+distress+syndrome%22&cntry=GB&age=12&draw=2&rank=17
NCT00789685	Safety, Tolerability and Preliminary Efficacy of FP-1201 in ALI and ARDS. Phase I/II	1. Clinically Significant Treatment Emergent Events [Time Frame: From first dose up until Day 28]<br>2. All Cause Mortality at Day 28 [Time Frame: 28 days following commencement of therapy]	2009	https://www.clinicaltrials.gov/ct2/show/NCT00789685?term=drug&type=Intr&cond=%22Acute+respiratory+distress+syndrome%22&cntry=GB&age=12&draw=2&rank=10
NCT00796419	Comparative Evaluation of Albumin and Starch Effects in Acute Lung Injury (ALI) (CEASE)	1. Change in Extravascular Lung Water (EVLW) [Time Frame: Baseline to Day 5 (120 h)]	2009	https://www.clinicaltrials.gov/ct2/show/NCT00796419?term=drug&type=Intr&cond=%22acute+respiratory+distress+syndrome%22&cntry=US&age=1&draw=4&rank=45

Ragel EJ, *et al. BMJ Open Respir Res* 2024; 10:e001525. doi: 10.1136/bmjresp-2022-001525

NCT00879606	Anti-TF Antibody (ALT-836) to Treat Septic Patients With Acute Lung Injury or Acute Respiratory Distress Syndrome	1. Safety profile of the study drug [Time Frame: Throughout the 28 days following treatment]<br>2. Number of ventilator-free days at Day 28 [Time Frame: Determined at Day 28]	2009	https://www.clinicaltrials.gov/ct2/show/NCT00879606?term=drug&type=Intr&cond=%22acute+respiratory+distress+syndrome%22&cntry=US&age=1&draw=4&rank=30
NCT00970606	STIP: Statin Trial for Influenza Patients (STIP)	1. Hospital Mortality to Day 28 or if Mortality is Not Different Between Groups, Time to Achieve Resolution of Respiratory Failure (e.g., Time to Unassisted Breathing in Survivors (Including Patient's Never Requiring Mechanical Ventilation). [Time Frame: 28 days]	2009	https://www.clinicaltrials.gov/ct2/show/NCT00970606?term=drug&type=Intr&cond=%22acute+respiratory+distress+syndrome%22&cntry=US&age=1&draw=4&rank=118
NCT00979121	Statins for Acutely Injured Lungs From Sepsis (SAILS)	1. Hospital Mortality to Day 60. [Time Frame: 60 days after randomization]	2010	https://www.clinicaltrials.gov/ct2/show/NCT00979121?term=drug&type=Intr&cond=%22acute+respiratory+distress+syndrome%22&cntry=US&age=1&draw=3&rank=72
NCT01050699	Sleep Intervention During Acute Lung Injury	1. Specific Aim 1: To assess the short-term effect of an α2 adrenergic agent on sleep quality in critically ill patients with ALI/ARDS. [Time Frame: 72 h]	2009	https://www.clinicaltrials.gov/ct2/show/NCT01050699?term=drug&type=Intr&cond=%22acute+respiratory+distress+syndrome%22&cntry=US&age=1&draw=4&rank=3
NCT01096771	The Effect of Intravenous Lipids on Lung Function in Acute Respiratory Distress Syndrome (ARDS)	1. Bronchoalveolar Lavage Fluid Interleukin-8 Concentrations [Time Frame: 96 h]	2010	https://www.clinicaltrials.gov/ct2/show/NCT01096771?term=drug&type=Intr&cond=%22acute+respiratory+distress+syndrome%22&cntry=US&age=1&draw=4

NCT01274481	Iloprost Effects on Gas Exchange and Pulmonary Mechanics	1. Arterial oxygenation [Time Frame: 30 min]	2011	https://www.clinicaltrials.gov/ct2/show/NCT01274481?term=drug&type=Intr&cond=%22acute+respiratory+distress+syndrome%22&cntry=US&age=1&draw=3&rank=80
NCT01335932	Ganciclovir/Valganciclovir for Prevention of CMV Reactivation in Acute Injury of the Lung and Respiratory Failure (GRAIL)	1. Serum IL-6 Level [Time Frame: Baseline and Day 14]	2011	https://www.clinicaltrials.gov/ct2/show/NCT01335932?term=drug&type=Intr&cond=%22acute+respiratory+distress+syndrome%22&cntry=US&age=1&draw=3&rank=94
NCT01438853	Effects of TNX-832 (Sunol cH36) in Subjects With Acute Lung Injury/Acute Respiratory Distress Syndrome	1. Safety assessed by number of adverse events, and changes in vital signs, ECGs, laboratory, coagulation and pulmonary function parameters. [Time Frame: Throughout the 4 weeks following treatment]<br>2. Composite of pharmacokinetics [Time Frame: predose; 15 and 30 min; 1, 4, 6, 12 and 24 hrs; 2, 3, 4, 5, 6, and 7 days, 2, 3, 4 weeks]	2004	https://www.clinicaltrials.gov/ct2/show/NCT01438853?term=drug&type=Intr&cond=%22acute+respiratory+distress+syndrome%22&cntry=US&age=1&draw=2
NCT01597635	The Safety, Tolerability, PK and PD of GSK2586881 in Patients With Acute Lung Injury	1. Heart Rate Assessments Up to Day 7 (Part B) [Time Frame: Up to Day 7]<br>2. Diastolic and Systolic Blood Pressure Assessments Up to Day 7 (Part B) [Time Frame: Up to Day 7]<br>3. Electrocardiogram (ECG) Parameters, Including PR, QRS, QT, and QTCU and RR Intervals Up to Day 7 (Part B) [Time Frame: Up to Day 7]<br>4. Hematology Parameters Basophils, Eosinophil, Lymphocytes, Monocytes, Total	2012	https://www.clinicaltrials.gov/ct2/show/NCT01597635?term=drug&type=Intr&cond=%22acute+respiratory+distress+syndrome%22&cntry=US&age=1&draw=4&rank=115

		Neutrophils, Platelet Count and White Blood Cell Count Up to Day 7 (Part B) [Time Frame: Up to Day 7]<br>5. Hematology Parameters Red Blood Cell Count and Reticulocyte Count Assessment Up to Day 7 (Part B) [Time Frame: Up to Day 7]<br>6. Hematology Parameter Hemoglobin and Mean Corpuscle Hemoglobin Concentration (MCHC) Assessment Up to Day 7 (Part B) [Time Frame: Up to Day 7]<br>7. Hematology Parameter Mean Corpuscle Volume (MCV) Assessment Up to Day 7 (Part B) [Time Frame: Up to Day 7]<br>8. Hematology Parameter Mean Corpuscle Hemoglobin (MCH) Assessment Up to Day 7 (Part B) [Time Frame: Up to Day 7]<br>9. Hematology Parameter Hematocrit Assessment Up to Day 7 (Part B) [Time Frame: Up to Day 7]<br>10. Clinical Chemistry Parameters Calcium, Chloride, Carbon Dioxide, Glucose, Potassium, Sodium, Urea/Blood Urea Nitrogen Assessment Up to Day 7 (Part B) [Time Frame: Up to Day 7]<br>11. Clinical Chemistry Parameters Direct Bilirubin, Total Bilirubin, Creatinine and Uric Acid Assessment Up to Day 7 (Part B) [Time Frame: Up to Day 7]<br>12. Clinical Chemistry Parameters Alkaline Phosphatase, Asparatate Amino Transferase, Alanine Amino Transferase, Gamma Glutamyl Transferase Assessment		

		Up to Day 7 (Part B) [Time Frame: Up to Day 7]<br>13. Clinical Chemistry Parameters Albumin and Total Protein Assessment Up to Day 7 (Part B) [Time Frame: Up to Day 7]<br>14. Number of Participant With Adverse Event (AE) and Serious Adverse Event (SAE) Assessment Up to Day 7 (Part A) [Time Frame: Up to Day 7]<br>15. Number of Par With AE and Serious Adverse Event (SAE) Assessment Up to Day 7 (Part B) [Time Frame: Up to Day 7]		
NCT02097641	Human Mesenchymal Stromal Cells For Acute Respiratory Distress Syndrome (START)	1. Numbers of Patients Occurred Pre-specified Infusion Associated Events Occurring Within 6 h of Study Infusion [Time Frame: 6 h]<br>2. Numbers of Patients Occurred Any Cardiac Arrest or Death Within 24 h of Study Infusion [Time Frame: 24 h]<br>3. Numbers of Patients Occurred Any Unexpected Severe Adverse Events (Including All-cause Deaths) [Time Frame: 12 months]	2014	https://www.clinicaltrials.gov/ct2/show/NCT02097641?term=drug&type=Intr&cond=%22acute+respiratory+distress+syndrome%22&cntry=US&age=1&draw=4&rank=146
NCT02106975	Vitamin C Infusion for Treatment in Sepsis Induced Acute Lung Injury (CITRIS-ALI)	1. Modified Change in Sequential Organ Failure Assessment (mSOFA) Score [Time Frame: 96 h]<br>2. C-Reactive Protein at Study Hours 0, 48, 96, 168 When Compared to Placebo [Time Frame: up to 168 h]<br>3. Thrombomodulin Protein at Study Hours 0, 48, 96, 168 When Compared to Placebo [Time Frame: Up to 168 h]	2014	https://www.clinicaltrials.gov/ct2/show/NCT02106975?term=drug&type=Intr&cond=%22acute+respiratory+distress+syndrome%22&cntry=US&age=1&draw=4&rank=101

NCT02425579	Safety Study of Inhaled Carbon Monoxide to Treat Acute Respiratory Distress Syndrome (ARDS)	1. Number of administration associated adverse events. [Time Frame: 60 Days if remains in the ICU]<br>2. Incidence of serious adverse events (SAEs). [Time Frame: 60 Days if remains in the ICU]	2015	https://www.clinicaltrials.gov/ct2/show/NCT02425579?term=drug&type=Intr&cond=%22acute+respiratory+distress+syndrome%22&cntry=US&age=1&draw=4&rank=34
NCT02509078	Reevaluation Of Systemic Early Neuromuscular Blockade (ROSE)	1. Hospital Mortality to Day 90 [Time Frame: 90 days after randomization]	2016	https://www.clinicaltrials.gov/ct2/show/NCT02509078?term=drug&type=Intr&cond=%22acute+respiratory+distress+syndrome%22&cntry=US&age=1&draw=4&rank=106
NCT02622724	Efficacy and Safety of FP-1201-lyo (Interferon Beta-1a) in Patients Having Acute Respiratory Distress Syndrome (ARDS) (INTEREST)	1. Composite Endpoint (VFDsurv; All-cause Mortality and Number of Days Free of Mechanical Ventilation) at Day 28 [Time Frame: Day 28]	2015	https://www.clinicaltrials.gov/ct2/show/NCT02622724?term=drug&type=Intr&cond=%22Acute+respiratory+distress+syndrome%22&cntry=GB&age=12&draw=2&rank=3
NCT02804945	Mesenchymal Stem Cells (MSCs) for Treatment of Acute Respiratory Distress Syndrome (ARD) in Patients With Malignancies	1. Adverse Events of Participants Treated with Allogeneic Human Mesenchymal Stem Cells (hMSCs) With Acute Respiratory Distress Syndrome (ARD) [Time Frame: 30 days]	2017	https://www.clinicaltrials.gov/ct2/show/NCT02804945?term=drug&type=Intr&cond=%22acute+respiratory+distress+syndrome%22&cntry=US&age=1&draw=2&rank=31
NCT03042143	Repair of Acute Respiratory Distress Syndrome by Stromal Cell Administration (REALIST) (COVID-19) (REALIST)	1. Oxygenation index (OI) [Time Frame: Day 7]<br>2. Incidence of Serious Adverse Events (SAEs) [Time Frame: 90 days]	2019	https://www.clinicaltrials.gov/ct2/show/NCT03042143?term=drug&type=Intr&cond=%22Acute+respiratory+distress+syndrome%22&cntry=GB&age=12&draw=2&rank=1

NCT03202394	Evaluation of Safety & Efficacy of BIO-11006 Inhalation Solution in Patients With ARDS	1. Incidence of treatment-emergent adverse events [Time Frame: 28 days]	2017	https://www.clinicaltrials.gov/ct2/show/NCT03202394?term=drug&type=Intr&cond=%22acute+respiratory+distress+syndrome%22&cntry=US&age=1&draw=4&rank=13
NCT03346681	N-Acetyl-cysteine in Early Acute Respiratory Distress Syndrome (NARDS)	1. Ventilator days [Time Frame: From time of intubation until one of predefined endpoints (up to 60 days)]	2018	https://www.clinicaltrials.gov/ct2/show/NCT03346681?term=drug&type=Intr&cond=%22acute+respiratory+distress+syndrome%22&cntry=US&age=1&draw=5&rank=53
NCT03376854	Pilot RCT of Therapeutic Hypothermia Plus Neuromuscular Blockade in COVID-19 Patients With ARDS (CHILL-pilot)	1. Targeted temperature compliance [Time Frame: Randomization through day 3]	2018	https://www.clinicaltrials.gov/ct2/show/NCT03376854?term=drug&type=Intr&cond=%22acute+respiratory+distress+syndrome%22&cntry=US&age=1&draw=4&rank=7
NCT03799874	Safety and Efficacy Study of Inhaled Carbon Monoxide to Treat Acute Respiratory Distress Syndrome (ARDS)	1. Primary Safety Outcome: Number of pre-specified administration-related adverse events. [Time Frame: 7 days]<br>2. Primary Efficacy Outcome: Change in Mitochondrial DNA (mtDNA) level from day 1 to day 5 [Time Frame: 5 days]	2019	https://www.clinicaltrials.gov/ct2/show/NCT03799874?term=drug&type=Intr&cond=%22acute+respiratory+distress+syndrome%22&cntry=US&age=1&draw=4&rank=49
NCT04115514	Treatment of ARDS With Instilled T3 (ARDS+T3)	1. Change in Extravascular Lung Water Index (EVLWI) [Time Frame: baseline, 1 h post T3 installation]<br>2. Change in Extravascular Lung Water Index (EVLWI) [Time Frame: baseline, 12 h post T3 installation]	2019	https://www.clinicaltrials.gov/ct2/show/NCT04115514?term=drug&type=Intr&cond=%22acute+respiratory+distress+syndrome%22&cntry=US&age=1&draw=4&rank=137

		3. Change in Extravascular Lung Water Index (EVLWI) [Time Frame: baseline, 24 h post T3 installation]		
NCT04345601	Mesenchymal Stromal Cells for the Treatment of SARS-CoV-2 Induced Acute Respiratory Failure (COVID-19 Disease)	1. Treatment-related serious adverse events (tSAEs) [Time Frame: 28 days post cell infusion]<br>2. Change in clinical status at day 14 [Time Frame: 14 days post cell infusion]	2021	https://www.clinicaltrials.gov/ct2/show/NCT04345601?term=drug&type=Intr&cond=%22acute+respiratory+distress+syndrome%22&cntry=US&age=1&draw=2&rank=11
NCT04351243	A Study to Assess the Efficacy and Safety of Gimsilumab in Subjects With Lung Injury or Acute Respiratory Distress Syndrome Secondary to COVID-19 (BREATHE)	1. Incidence of Mortality [Time Frame: Day 43]	2020	https://www.clinicaltrials.gov/ct2/show/NCT04351243?term=drug&type=Intr&cond=%22acute+respiratory+distress+syndrome%22&cntry=US&age=1&draw=4&rank=104
NCT04355728	Use of UC-MSCs for COVID-19 Patients	1. Number of Participants With Pre-Specified Infusion Associated Adverse Events [Time Frame: 6 and 24 h]<br>2. Number of Subjects With Serious Adverse Events by 31 Days After First Infusion [Time Frame: 31 days]<br>3. Percentage of Participants Experiencing Serious Adverse Events (SAEs) Through Study Day 90 [Time Frame: 90 days]<br>4. Number of Adverse Events (AEs) and Serious Adverse Events (SAEs) [Time Frame: 90 days]<br>5. Number of Adverse Events (AEs) and Serious Adverse Events (SAEs) by Severity [Time Frame: 90 days]	2020	https://www.clinicaltrials.gov/ct2/show/NCT04355728?term=drug&type=Intr&cond=%22acute+respiratory+distress+syndrome%22&cntry=US&age=1&draw=3&rank=56

		6. Subjects With Adverse Events and Serious Adverse Events by Severity [Time Frame: 90 days]<br>7. Number of Adverse Events and Serious Adverse Events by Relatedness to Treatment [Time Frame: 90 days]<br>8. Subjects With Adverse Events by Relatedness to Treatment [Time Frame: 90 days]		
NCT04357730	Fibrinolytic Therapy to Treat ARDS in the Setting of COVID-19 Infection	1. PaO_2/FiO_2 Change (Increase) From Pre-to-post Intervention [Time Frame: at 48 h post randomization]	2020	https://www.clinicaltrials.gov/ct2/show/NCT04357730?term=drug&type=Intr&cond=%22acute+respiratory+distress+syndrome%22&cntry=US&age=1&draw=3&rank=87
NCT04360096	Inhaled ZYESAMI™ (Aviptadil Acetate) for the Treatment of Severe COVID-19 (AVICOVID-2)	1. Progression to Respiratory Failure [Time Frame: 28 days]	2021	https://www.clinicaltrials.gov/ct2/show/NCT04360096?term=drug&type=Intr&cond=%22acute+respiratory+distress+syndrome%22&cntry=US&age=1&draw=5
NCT04369469	Efficacy and Safety Study of IV Ravulizumab in Patients With COVID-19 Severe Pneumonia	1. Survival (based on all-cause mortality) at Day 29 [Time Frame: Baseline, Day 29]	2020	https://www.clinicaltrials.gov/ct2/show/NCT04369469?term=drug&type=Intr&cond=%22acute+respiratory+distress+syndrome%22&cntry=US&age=1&draw=3&rank=71
NCT04371393	MSCs in COVID-19 ARDS	1. Number of all-cause mortality [Time Frame: 30 days]	2020	https://www.clinicaltrials.gov/ct2/show/NCT04371393?term=drug&type=Intr&cond=%22acute+respiratory+distress+syndrome%22&cntry=US&age=1&draw=3

NCT04384445	Zofin (Organicell Flow) for Patients With COVID-19	1. Incidence of any infusion associated adverse events [Time Frame: 60 Days]<br>2. Incidence of Severe Adverse Events [Time Frame: 60 Days]	2020	https://www.clinicaltrials.gov/ct2/show/NCT04384445?term=drug&type=Intr&cond=%22acute+respiratory+distress+syndrome%22&cntry=US&age=1&draw=3
NCT04389671	The Safety and Preliminary Tolerability of Lyophilized Lucinactant in Adults With Coronavirus Disease 2019 (COVID-19)	1. Evaluate the safety and feasibility of Lucinactant surfactant replacement therapy (SRT) in treating COVID-19 [Time Frame: (OI) through 12 h post dosing and other physiological and outcome measurements through 24 h or through Day 30]<br>2. Oxygen index (OI) [Time Frame: Baseline through 12 h post initiation of dosing]	2020	https://www.clinicaltrials.gov/ct2/show/NCT04389671?term=drug&type=Intr&cond=%22acute+respiratory+distress+syndrome%22&cntry=US&age=1&draw=4&rank=119
NCT04397510	Nebulized Heparin for the Treatment of COVID-19 Induced Lung Injury	1. Mean daily PaO_2 to FiO_2 ratio [Time Frame: 10 days]	2020	https://www.clinicaltrials.gov/ct2/show/NCT04397510?term=drug&type=Intr&cond=%22acute+respiratory+distress+syndrome%22&cntry=US&age=1&draw=5
NCT04399889	hCT-MSCs for COVID19 ARDS	1. Safety of the Investigational Product-Infusion Reactions [Time Frame: 24 h]<br>2. Safety of the Investigational Product-delayed reactions [Time Frame: 28 days]<br>3. Safety of the Investigational Product-formation of anti-HLA antibodies [Time Frame: 28 days]	2020	https://www.clinicaltrials.gov/ct2/show/NCT04399889?term=drug&type=Intr&cond=%22acute+respiratory+distress+syndrome%22&cntry=US&age=1&draw=4&rank=111
NCT04401527	Treatment of Lung Injury From COVID-19 Infection With Intravenous Sodium Nitrite	1. Survival with Unassisted Breathing [Time Frame: Day 28]	2020	https://www.clinicaltrials.gov/ct2/show/NCT04401527?term=drug&type=Intr&cond=%22acute+respiratory+distr

				ess+syndrome%22&cntry=US&age=1&draw=4&rank=5
NCT04402060	A Study of APL-9 in Adults With Mild to Moderate ARDS Due to COVID-19	1. Number of Subjects Who Experienced Treatment-emergent Adverse Events (TEAEs) and Serious TEAEs [Time Frame: From the first dose of study drug and up to 30 (+7) days after the last dose of study drug. Part 1: Day 1 up to Day 44; Part 2: Day 1 up to Day 58]	2020	https://www.clinicaltrials.gov/ct2/show/NCT04402060?term=drug&type=Intr&cond=%22acute+respiratory+distress+syndrome%22&cntry=US&age=1&draw=3&rank=100
NCT04412057	Clinical Trial to Evaluate CERC-002 in Adults With COVID-19 Pneumonia and Acute Lung Injury	1. Number of Subjects Alive and Free of Respiratory Failure [Time Frame: Baseline to Day 28]	2020	https://www.clinicaltrials.gov/ct2/show/NCT04412057?term=drug&type=Intr&cond=%22acute+respiratory+distress+syndrome%22&cntry=US&age=1&draw=3&rank=68
NCT04417036	This Study Collects Information on the Safety of Inhaled Pegylated Adrenomedullin (PEG-ADM), How the Drug is Tolerated and How it Affects Patients Suffering From a Type of Lung Failure That Cause Fluid to Build up in the Lungs Making Breathing Difficult (ARDS)	1. VFS in Part B participants [Time Frame: At Day 28]	2020	https://www.clinicaltrials.gov/ct2/show/NCT04417036?term=drug&type=Intr&cond=%22Acute+respiratory+distress+syndrome%22&cntry=GB&age=12&draw=2&rank=2
NCT04468971	REgulatory T Cell infuSion fOr Lung Injury Due to COVID-19 PnEumonia (RESOLVE)	1. Regimen related ≥ grade 3 toxicity within 48 h of first infusion [Time Frame: 48 h]<br>2. 28-day treatment success, defined as S28 [Time Frame: 28 days]	2020	https://www.clinicaltrials.gov/ct2/show/NCT04468971?term=drug&type=Intr&cond=%22acute+respiratory+distress+syndrome%22&cntry=U

				S&age=1&draw=4&rank=134
NCT04478071	Vadadustat for the Prevention and Treatment of Acute Respiratory Distress Syndrome (ARDS) in Hospitalized Patients With Coronavirus Disease 2019 (COVID-19)	1. Number of participants who are classified 8 (dead), 7 (hospitalized, on invasive mechanical ventilation or ECMO), or 6 (hospitalized, on non-invasive ventilation or high flow oxygen devices) on the NIAID ordinal scale [Time Frame: day 14]	2020	https://www.clinicaltrials.gov/ct2/show/NCT04478071?term=drug&type=Intr&cond=%22acute+respiratory+distress+syndrome%22&cntry=US&age=1&draw=5
NCT04482621	Decitabine for Coronavirus (COVID-19) Pneumonia - Acute Respiratory Distress Syndrome (ARDS) Treatment: DART Trial (DART)	1. Proportion of patients who are alive and free of respiratory failure at day 28 [Time Frame: From the day of randomization to day 28]	2020	https://www.clinicaltrials.gov/ct2/show/NCT04482621?term=drug&type=Intr&cond=%22acute+respiratory+distress+syndrome%22&cntry=US&age=1&draw=4&rank=37
NCT04482699	RAPA-501-Allo Therapy of COVID-19-ARDS	1. Dose-Limiting Toxicity (DLT) [Time Frame: Through day 7.]<br>2. Mortality Rate [Time Frame: 30 days after the first infusion of allogeneic RAPA-501 cells.]	2020	https://www.clinicaltrials.gov/ct2/show/NCT04482699?term=drug&type=Intr&cond=%22acute+respiratory+distress+syndrome%22&cntry=US&age=1&draw=4&rank=127
NCT04490486	Umbilical Cord Tissue (UC) Derived Mesenchymal Stem Cells (MSCs) Versus Placebo to Treat Acute Pulmonary Inflammation Due to COVID-19	1. Percent of participants with treatment related Serious Adverse Events (SAE) [Time Frame: 12 months]	2022	https://www.clinicaltrials.gov/ct2/show/NCT04490486?term=drug&type=Intr&cond=%22acute+respiratory+distress+syndrome%22&cntry=US&age=1&draw=2&rank=42
NCT04502433	Poractant Alfa - Curosurf and SARS-COV-19 ARDS (Covid-19)	1. Number of days alive and ventilator-free days [Time Frame: up to 21 days]	2021	https://www.clinicaltrials.gov/ct2/show/NCT04502433?term=drug&type=Intr&cond=%22acute+respiratory+distr

				ess+syndrome%22&cntry=US&age=1&draw=4&rank=123
NCT04530604	Defibrotide Therapy for SARS-CoV2 (COVID-19) Acute Respiratory Distress Syndrome (ARDS)	1. Number of major hemorrhagic complications within 14 days of initiation of treatment [Time Frame: 14 days]	2020	https://www.clinicaltrials.gov/ct2/show/NCT04530604?term=drug&type=Intr&cond=%22acute+respiratory+distress+syndrome%22&cntry=US&age=1&draw=4&rank=6
NCT04537806	A Study of Brexanolone for Acute Respiratory Distress Syndrome Due to COVID-19	1. Percentage of Participants Who are Alive and Free of Respiratory Failure at Day 28 [Time Frame: Day 28]	2020	https://www.clinicaltrials.gov/ct2/show/NCT04537806?term=drug&type=Intr&cond=%22acute+respiratory+distress+syndrome%22&cntry=US&age=1&draw=4
NCT04545424	Trial of Therapeutic Hypothermia in Patients With ARDS (CHILL)	1. 28-day ventilator-free days (VFDs) [Time Frame: Calculated at study day 28 or death (whichever occurs first)]	2021	https://www.clinicaltrials.gov/ct2/show/NCT04545424?term=drug&type=Intr&cond=%22acute+respiratory+distress+syndrome%22&cntry=US&age=1&draw=4&rank=41
NCT04565249	Evaluation of the Safety, Tolerability, and Pharmacokinetics of PLN-74809 in Participants With Acute Respiratory Distress Syndrome (ARDS) Associated With at Least Severe COVID-19	1. Number of participants with treatment-related adverse events and laboratory abnormalities, assessed by CTCAE V5.0 [Time Frame: Up to 90 days]	2020	https://www.clinicaltrials.gov/ct2/show/NCT04565249?term=drug&type=Intr&cond=%22acute+respiratory+distress+syndrome%22&cntry=US&age=1&draw=4&rank=109
NCT04582201	An Experiment to Evaluate the Safety of agenT-797 in	1. Number Of Participants With Treatment-emergent Adverse Events [Time Frame: Baseline through Month 6]	2020	https://www.clinicaltrials.gov/ct2/show/NCT04582201?term=drug&type=Intr&cond=

	COVID-19 Patients With Severe Difficulty Breathing.	2. Number Of Participants With Dose-limiting Toxicities [Time Frame: Baseline through Month 6]		%22acute+respiratory+distress+syndrome%22&cntry=US&age=1&draw=2&rank=2
NCT04616586	SILtuximab in Viral ARds (SILVAR) Study (SILVAR)	1. 28-day all-cause mortality [Time Frame: Day 28]	2020	https://www.clinicaltrials.gov/ct2/show/NCT04616586?term=drug&type=Intr&cond=%22acute+respiratory+distress+syndrome%22&cntry=US&age=1&draw=4
NCT04629105	Regenerative Medicine for COVID-19 and Flu-Elicited ARDS Using Lomecel-B (RECOVER)	1. Incidence of Treatment-Emergent Serious Adverse Events [Time Frame: Within 4 weeks after treatment]<br>2. Number of Participants with Abnormal Clinical Significant Laboratory Values in Hematology. [Time Frame: Baseline to 6 Months]<br>3. Number of Participants with Changes in Echocardiography Overall Assessment [Time Frame: Baseline to 6 Months]<br>4. Number of Participants with Changes to overall assessment of Electrocardiogram [Time Frame: Baseline to 6 Months]<br>5. Time to recovery of $Sp0_2$ [Time Frame: Baseline to 6 Months]<br>6. Number of Participants with Abnormal Clinical Significant Lab Values in the Blood Chemistry testing. [Time Frame: Baseline to 6 months]<br>7. Number of Participants with Abnormal Clinical Significant Lab Values in the Coagulation. [Time Frame: Baseline to 6 months]	2020	https://www.clinicaltrials.gov/ct2/show/NCT04629105?term=drug&type=Intr&cond=%22acute+respiratory+distress+syndrome%22&cntry=US&age=1&draw=2&rank=32

		8. Number of Participants with Abnormal Clinical Significant Lab Values in the Urinalysis [Time Frame: Baseline to 6 months]		
NCT04725110	Direct Topical Lung T3 Treatment to Improve Outcome & Sequelae of COVID-19 Acute Respiratory Distress Syndrome	1. Change Extravascular Lung Water Index [Time Frame: 1 h]	2022	https://www.clinicaltrials.gov/ct2/show/NCT04725110?term=drug&type=Intr&cond=%22acute+respiratory+distress+syndrome%22&cntry=US&age=1&draw=4&rank=107
NCT04750278	A Phase 2/3, Randomized, Double Blind, Placebo Controlled, Multicenter Study to Evaluate the Efficacy and Safety of FP-025 in Patients With Severe to Critical COVID 19 With Associated Acute Respiratory Distress Syndrome (ARDS)	1. The proportion of patients alive and not requiring non-invasive or invasive ventilation [Time Frame: Day 28]	2021	https://www.clinicaltrials.gov/ct2/show/NCT04750278?term=drug&type=Intr&cond=%22acute+respiratory+distress+syndrome%22&cntry=US&age=1&draw=3
NCT04778059	Safety and Efficacy of USB002 for Respiratory Distress Due to COVID-19	1. Number of participants with treatment emergent adverse events (TEAE) [Time Frame: Day 1 to Day 70 (or date of final measurement, if sooner)]	2021	https://www.clinicaltrials.gov/ct2/show/NCT04778059?term=drug&type=Intr&cond=%22acute+respiratory+distress+syndrome%22&cntry=US&age=1&draw=4&rank=128
NCT04798716	The Use of Exosomes for the Treatment of Acute Respiratory Distress Syndrome or Novel Coronavirus Pneumonia	1. Measure and report the number of participants with treatment-related-adverse events as assessed by CTCAE v4.0; for patients receiving ARDOXSO™, perinatal	2021	https://www.clinicaltrials.gov/ct2/show/NCT04798716?term=drug&type=Intr&cond=%22acute+respiratory+distr

	Caused by COVID-19 (ARDOXSO)	MSC-derived exosome therapy. [Time Frame: 90 Days]<br>2. Tabulate and report the number of IMV days for patients receiving ARDOXSO™ perinatal MSC-derived exosome therapy. [Time Frame: 90 Days]		ess+syndrome%22&cntry=US&age=1&draw=3&rank=76
NCT04870125	Safety Study of Inhaled Carbon Monoxide to Treat Sepsis-Induced Acute Respiratory Distress Syndrome (ARDS)	1. Primary Safety Outcome: Number of pre-specified administration-related adverse events (AEs). [Time Frame: 7 days]<br>2. Accuracy of the Coburn-Forster-Kane (CFK) equation-based personalized iCO dosing algorithm to achieve a COHb level of 6–8% [Time Frame: day 1, day 2, and day 3]	2022	https://www.clinicaltrials.gov/ct2/show/NCT04870125?term=drug&type=Intr&cond=%22acute+respiratory+distress+syndrome%22&cntry=US&age=1&draw=4&rank=24
NCT04905836	Study of Allogeneic Adipose-Derived Mesenchymal Stem Cells for Treatment of COVID-19 Acute Respiratory Distress	1. All-cause mortality rate at Day 28 [Time Frame: Baseline through Day 28]<br>2. Incidence of all adverse events (AEs) (safety) [Time Frame: Baseline through study completion at Day 90]<br>3. Incidence of treatment-emergent adverse events (safety) [Time Frame: Baseline through study completion at Day 90]<br>4. Incidence of severe adverse events (safety) [Time Frame: Baseline through study completion at Day 90]<br>5. Incidence of infusion-related adverse events (safety) [Time Frame: Baseline to Hour 4]	2021	https://www.clinicaltrials.gov/ct2/show/NCT04905836?term=drug&type=Intr&cond=%22acute+respiratory+distress+syndrome%22&cntry=US&age=1&draw=4

Table ESI1. A complete list of 66 CTs with primary outcome(s), start year and web link.

Ragel EJ, *et al. BMJ Open Respir Res* 2024; 10:e001525. doi: 10.1136/bmjresp-2022-001525

Intervention	ARDS	CARDS
Anti-tissue factor	**NCT00879606, NCT01438853:** ALT-836 was deemed safe and tolerable; no disease outcome measures were assessed<br>→ *Unknown effect*	No trials<br>→ *Unknown effect*
CD14 inhibitor (IC14)	NCT00233207: study terminated<br>→ *Unknown effect*	No trials<br>→ *Unknown effect*
T-cell therapy	No trials<br>→ *Unknown effect*	**NCT04582201:** agenT-797 trial ongoing<br>**NCT04482621:** Decitabine trial ongoing<br>**NCT04482699:** RAPA-501 data not yet available<br>**NCT04468971:** Treg infusion trial ongoing<br>→ *Unknown effect*
Antifibrotics	No trials<br>→ *Unknown effect*	**NCT04565249:** PLN-74809 trial was discontinued<br>→ *Unknown effect*
Neuromuscular blocking agents	**NCT04545424:** hypothermia data not yet available<br>**NCT02509078:** Cisatracurium besylate infusion did not reduce mortality at 90 days and increased the incidence of adverse cardiovascular events<br>→ *No effect*	**NCT03376854:** Hypothermia and neuromuscular blockade trial was withdrawn; no data available<br>→ *Unknown effect*
Surfactants	**NCT00682500:** Calfactant was not associated with improved survival, or oxygenation and was frequently	**NCT04502433:** Curosurf® trial terminated

Ragel EJ, *et al. BMJ Open Respir Res* 2024; 10:e001525. doi: 10.1136/bmjresp-2022-001525

	associated with transient hypoxia and hypotension<br>**NCT00215553:** KL_4 surfactant trial terminated; no data available<br>**NCT00742482:** HL10 did not improve outcome and may increase mortality and adverse effects<br>→ *No effect*	NCT04389671: Final Lucinactant data not yet available<br>→ *Unknown effect*
Granulocyte-macrophage colony-stimulating factor	**NCT00201409:** No change in ventilator-free days, 28-day mortality or organ failure-free days with treatment<br>→ *No effect*	No trials<br>→ *Unknown effect*
Granulocyte-macrophage colony-stimulating factor inhibitor (gimsilumab)	No trials<br>→ *Unknown effect*	**NCT04351243:** no improvement in mortality or other key clinical outcomes with treatment<br>→ *No effect*
C3 inhibitor (APL-9)	No trials<br>→ *Unknown effect*	**NCT04402060:** no effect on any outcome measure assessed<br>→ *No effect*

Table ESI2. A comparison of therapeutic interventions that have had unknown or no effect in ARDS versus CARDS patients in CTs with published results. NCT numbers of the corresponding CT are listed where appropriate, and remarks about the intervention for the two conditions are stated; effectiveness is defined as improvements in patient outcomes and/or clinical measures.

Ragel EJ, *et al. BMJ Open Respir Res* 2024; 10:e001525. doi: 10.1136/bmjresp-2022-001525
